# A Preliminary Study on Realizing Human–Robot Mental Comforting Dialogue via Sharing Experience Emotionally

**DOI:** 10.3390/s22030991

**Published:** 2022-01-27

**Authors:** Changzeng Fu, Qi Deng, Jingcheng Shen, Hamed Mahzoon, Hiroshi Ishiguro

**Affiliations:** 1 Graduate School of Engineering Science, Osaka University, Toyonaka 560-8531, Japan; mahzoon@irl.sys.es.osaka-u.ac.jp (H.M.); ishiguro@irl.sys.es.osaka-u.ac.jp (H.I.); 2 Interactive Robot Research Team, Robotics Project, RIKEN, Kyoto 619-0237, Japan; 3 Graduate School of Intercultural Studies, Kobe University, Kobe 657-8501, Japan; dengqixq123@gmail.com; 4 Graduate School of Information Science, Osaka University, Suita 565-0871, Japan; jc-shen@ist.osaka-u.ac.jp

**Keywords:** emotional interaction, experience sharing, human–robot conversation

## Abstract

Mental health issues are receiving more and more attention in society. In this paper, we introduce a preliminary study on human–robot mental comforting conversation, to make an android robot (ERICA) present an understanding of the user’s situation by sharing similar emotional experiences to enhance the perception of empathy. Specifically, we create the emotional speech for ERICA by using CycleGAN-based emotional voice conversion model, in which the pitch and spectrogram of the speech are converted according to the user’s mental state. Then, we design dialogue scenarios for the user to talk about his/her predicament with ERICA. In the dialogue, ERICA shares other people’s similar predicaments and adopts a low-spirit voice to express empathy to the interlocutor’s situation. At the end of the dialogue, ERICA tries to encourage with a positive voice. Subsequently, questionnaire-based evaluation experiments were conducted with the recorded conversation. In the questionnaire, we use the Big Five scale to evaluate ERICA’s personality. In addition, the perception of emotion, empathy, and encouragement in the dialogue are evaluated. The results show that the proposed emotional expression strategy helps the android robot better present low-spirit emotion, empathy, the personality of extroversion, while making the user better feel the encouragement.

## 1. Introduction

Emotional human–robot interaction (HRI) has received a lot of attention in recent decades, with some applications in areas such as child care [1], mental therapy [2], personality presentation [3], and so on. These studies have demonstrated the importance of emotional expression in human–robot interactions to enhance engagement and improve the perception of intelligence [4,5]. So far, most of the research related to emotional interaction has focused on non-verbal behavior in expressing robot’s emotions, which does not necessitate a high level understanding ability on the part of the robot with respect to the interaction context. However, in some scenarios, robots may be required to display both semantic understanding and emotion comprehension to interact with humans verbally and non-verbally, for example, the emotional comfort conversation [6].

Comforting conversation can be regarded as an emotional supporting behavior that offers reassurance, encouragement, and compassion [7]. In human–human interactions, comforting behavior requires verbal expressions of empathy that present an emotional understanding of other people’s feelings from their perspective to convey motivational content for encouragement [8,9,10]. In other words, the realization of the comforting dialogue requires the robot to convey semantic understanding, emotional understanding, and empathy. These abilities need to be expressed in some way during the interaction, which is difficult to achieve by simply using some constructed models or features, such as a chatbot that has an emotional response [11] or personal information disclosure chatbot [12]. The former does not allow robots to demonstrate that it can understand other people’s views, while the latter does not allow robots to demonstrate that it can experience the user’s feelings. In this paper, the focus is on the expression of the robot in comforting conversations to demonstrate the robot’s ability to understand, feel, and empathize.

To this end, the concept of person-centered message was adopted. According to some psychological studies [13], person-centered messages recognize and adapt to the emotional, subjective, and relational characteristics of a given situation. High et al. [14] discussed the effectiveness of the person-centered message in social support. They found that people receiving the person-centered message are more likely to experience tangible affective benefits. Moreover, they associated the person-centered message with people’s experience of predicament. In the human–robot interaction field, some studies propose that robots should be allowed to share people-related experiences during interactions [15,16,17] to present perspective [18,19,20]. They found that the experience sharing strategy can help the robot display mind-attribution, intelligence, while maintaining the long-term interaction to better build the human–robot relationship. However, the effect of the combination of experience and emotion in human–robot interactions is not discussed. Therefore, this work mainly explores the emotion factor with an experience-sharing strategy in human–robot comforting dialogues.

In this study, the possibility of using a robot for comforting dialogue is explored as follows:

- We investigate how humans react to other’s emotions in human–human interactions (HHI) based on the IEMOCAP [21] and MELD [22] datasets;
- We build an emotional voice conversion model to obtain ERICA’s emotional voice;
- We let an android robot, ERICA, provide scenario-based comforts to users by expressing corresponding emotions in verbal behavior;
- We construct people-centered messages in robot utterances by sharing related experiences/situations of other people in historical human–robot interactions;
- We adopt the questionnaire-based evaluation with a Likert scale to examine the effectiveness of emotional experience sharing in comforting dialogues;
- In addition, we evaluate ERICA’s personality based on BIG FIVE personality traits [23].

The rest of this chapter is organized as follows. Related works are introduced in Section 2. In Section 3, the emotional feedback provided in human–human interactions is investigated. In Section 4, our method is described. In Section 5, the setting of the experiment and results are demonstrated. Some limitations and future work are presented in Section 6. Finally, the findings are discussed prior to concluding remarks.

## 2. Related Works

### 2.1. Human–Robot Comforting Interaction

The emotional robot has been explored in the field of human–robot interaction; however, there are few studies on comforting. Researchers are expected to relieve users’ stress, dissipate anxiety, and provide comfort through robots via emotional interactions. Wada et al. [24] and Jagkapong et al. [25] used a seal robot for the therapy of elders suffering from dementia and autistic children. The experimental results showed that robots could improve the therapy of elders and increase the communication, motivation of autistic children. Later, Raihah et al. [26] used the same robot to investigate the effects of interaction between people who have not been diagnosed with any mental health disorders along with a social robot to assess psychophysiological stress responses. However, the above studies focused on non-verbal behavior.

To extend this scenario into verbal emotional support and interaction, Silvia et al. [27] used a social robot to interact with children to apply distraction strategies, to reduce anxiety during vaccination. The authors developed some dialogue strategies, including small talk, music sharing, and questioning. Results showed that the distraction strategies were able to reduce fear and anxiety while increasing happiness. Baecker et al. [28] implemented a robot to help otherwise-healthy older adults living in social isolation and loneliness. The author developed a dialogue function that listened to the user’s predicament to start a conversation on that. Their experimental results showed that the proposed method was able to provide emotional support through the designed conversation strategy. However, emotional expressions were not included in their study, and no effective strategy was considered for the expression of empathy by robots.

### 2.2. Robot’s Emotion in Human–Robot Interaction

To enhance the engagement of users in social human–robot interactions, it is important that robots be capable of reacting to the emotions expressed by the human agent with affective expressions [29]. According to Paiva et al. [30], emotional robots should perceive the ambient environment (users’ mental state in this work) and emotionally respond to it. This procedure can be placed in the affective loop. Affective loop is the interactive process in which the user first expresses an emotion with interactions. The robot, in turn, responds by generating emotional expressions, causing the user to respond and gradually feel more and more engaged with the system. In this loop, the robot requires emotion detection ability and emotion expression ability. With the development of deep learning, the emotion detection can somehow be easily addressed [31,32,33]. Regarding the emotional interaction of social robots, numerous works have attempted to address this in recent decades. One of the most commonly used methods is mimicking the user’s affective state [34,35]. The results of those studies suggest that the mimicking strategy makes the robots’ responses more appropriate to the interaction context than without mimicking. Another is the perspective-taking strategy. According to [36], there are at least two dimensions of perspective-taking, perceptual and conceptual. Perceptual perspective-taking is defined as the ability to understand how another person experiences things through their senses. Conceptual perspective-taking is defined as the ability to comprehend and take on the viewpoint of another person’s psychological experience (e.g., thoughts, feelings). To present such abilities in human–robot conversation, the addressee (robot) should use their knowledge of the user’s situation. Experience sharing can be a potential way to allow robots to demonstrate “I learnt/obtained the knowledge from other people in the past conversations”, expressing the viewpoint of the user’s psychological experience indirectly. To date, the perspective-taking ability is considered an essential ability for empathic robots [18,19,20]. These studies discovered that the robot’s perspective-taking behavior is perceived as friendlier and facilitates the communication during human–robot interactions. However, these studies did not explore how to leverage emotional expression in human–robot comforting. Additionally, investigations on the effects of the combination of emotions and experience sharing (perspective-taking) are lacking.

### 2.3. Audio Modality Emotional Expression for Robots

Voice synthesis technology has made it easier for robots to communicate with their human counterparts through speech. A lot of studies have attempted to use modern speech synthesizers to convey robots’ emotions. Crumpton et al. [37] and Nass et al. [38] generated the robots’ emotional speech by manually modifying the vocal prosody. Roehling et al. [39] investigated the text-to-speech (TTS) systems to synthesize emotional speech for robots, such as DECtalk, Festival, and OpenMARY. However, these toolkits are created for English.

Considering the Japanese emotional TTS, Lee et al. [40] adopted AItalk, a Japanese TTS engine that generates emotional speech, to endow robot NAO with affective expression ability. They also controlled the emotions with manual modification of acoustic features. However, the voices in AItalk are specific to a few avatars. Considering that ERICA’s voice should be based on her own characteristics, ERICA’s features should be maintained when enhancing emotional characteristics. Therefore, the CycleGAN-based emotional voice conversion model was trained to convert the neutral voice generated by ERICA’s TTS to an emotional one.

## 3. Investigation of Human–Human Interaction

To observe the human emotional feedback in human–human interactions according to the interlocutor’s emotional status, we conducted an investigation based on the IEMOCAP [21] and MELD [22] datasets. The strategy for robot’s emotional responses can be appropriately designed based on the findings.

The used datasets can be briefly summarized as follows: IEMOCAP dataset contains videos of two-way conversations with 10 speakers. The actors performed selected emotional scripts and also improvised hypothetical scenarios designed to elicit specific types of emotions. In this study, 5683 samples from happy, sad, frustrated, surprise, angry, excited, and neutral emotions were selected for analysis. Fear and disgust were not included because of the small number of samples. The MELD dataset is a multi-party conversational dataset collected from an American TV series. The 9989 samples selected for later analysis consisted of disgust, joy, neutral, anger, fear, sadness, and neutral.

Table 1 and Table 2 present the analysis results. It is observed that the interlocutor often responds with the same or similar emotion of the speaker in the IEMOCAP dataset. In the MELD dataset, the interlocutor prefers to use neutral emotion to respond to the speaker’s emotion. In addition, it is also observed that, for a high percentage of the cases, the interlocutors respond to the speaker using the same emotion. Based on the above results, it is reasonable to have the robot respond using the same emotion or neutral emotion as the speaker. The results also imply that the speaker and interlocutor tend to be emotionally aligned. For example, if the speaker is happy, the interlocutor is happy; if the speaker is sad, the interlocutor is sad.

According to the aforementioned facts in HHI, as well as the findings of mimicking strategy [34,35], a mechanism for letting the robot exert some influence on the user with positive emotions should be implemented with the purpose of providing comfort to the subject, transforming their mental status. In this study, sadness was defined as a negative emotion and happiness as a positive emotion instead of using the two-dimensional emotional model (negative-positive and low–high arousal). It was assumed that the participants in the experiment could better understand the defined negative and positive emotions without the arousal dimension, which is also commonly mentioned in previous comfort-related research [41,42].

## 4. Method

### 4.1. Emotional Voice

**Figure 1:**
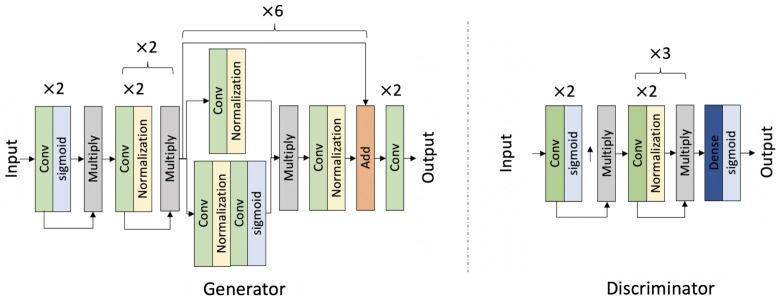
Neural networks for CycleGAN-based emotional voice conversion model.

The generation of ERICA’s voice was originally based on a text-to-speech system, which can only generate the neutral voice. To create the emotional voice for ERICA, in this study, we built an emotional voice conversion model. To this end, a Japanese emotional speech dataset [43] that contains happy, angry, sad, and neutral utterances was used. Each category of this dataset has 1070 utterances in total. Sad emotion was regarded as low-spirit, and happy as a positive voice. Because the sample of the Japanese emotional speech dataset was non-paralleled, a model that could be trained on non-paralleled data was required to realize the emotional voice conversion. To this end, we adopted the CycleGAN framework structure, which had been widely demonstrated to have excellent performance on non-paralleled data [44,45,46]. Figure 1 presents the structure of the CycleGAN model. The generator first employed the convolution layer with a shortcut by multiplying the output of the convolution layer and activation function. The convolution block was repeated twice. Then, a similar block with the normalization layer was used. After that, a residual convolutional block was employed and repeated six times, followed by a final convolution layer. The discriminator module used the same convolutional block as the generator. Then, a dense layer and a sigmoid function were used to output the real or fake label.

During the training phase, the CycleGAN model was trained with (neutral, low-spirit) and (neutral, positive) combinations to realize the emotion conversion. The F0 feature and spectrogram were separately converted to assure that both prosody and phase were transformed. Regarding the extraction of the F0 feature, we adopted the continuous wavelet transform (CWT) to decompose the F0 contour with 10 temporal scales as performed in previous research [45,47]. Equations (1) and (2) demonstrate the calculations, where F0(x) indicates the input F0 signal; ϕ denotes the Mexican hat mother wavelet; τ=5 ms, and i∈[1,10] are one octave apart.

(1) $$W\left(F0\right)\left(\tau ,t\right)=\tau^{-1/2}\int F0\left(x\right)\phi \left(\frac{x-t}{\tau }\right)dx$$

(2) $$F0\left(t\right)=\sum_{i=1}^{10}W_{i}\left(F0\right)\left(t\right){\left(i+2.5\right)}^{-5/2}$$

To extract the spectrogram from speech, cheaptrick [48] was employed. As presented in Equation (3), this technology first smooths the spectrogram by using a window with a width of $2w_{0}/3$.

(3) $$\begin{array}{ccc} P(w) & =\frac{3}{2w_{0}}\int_{-w_{0}/3}^{w_{0}/3}P\left(w+\sigma \right)d\sigma w_{0} & =2\pi /\tau_{0} \end{array}$$

Then, liftering was applied to the quefrency domain to mitigate the fluctuations:

(4) $$\begin{array}{cc} \; & P\left(w\right)=exp(F\left[l_{s}\left(\tau \right)l_{q}\left(\tau \right)p_{s}\left(\tau \right)\right])\\ \; & l_{s}\left(\tau \right)=\frac{sin(\pi F0\tau )}{\pi F0\tau }\\ \; & l_{q}\left(\tau \right)=q_{0}+2q_{1}cos\left(2\pi \tau /\tau_{0}\right)\\ \; & p_{s}\left(\tau \right)=F^{-1}\left[log\left(P\left(w\right)\right)\right] \end{array}$$

where F and $F^{-1}$ stand for the Fourier transform and its inverse, respectively; $l_{s}\left(\tau \right)$ indicates the liftering function to smooth the signal; $l_{q}\left(\tau \right)$ stands for the liftering function for spectral recovery; $p_{s}\left(\tau \right)$ represents the cepstrum of P(w); $q_{0}$ and $q_{1}$ are set to 1.18 and −0.09 as discussed in [48].

Furthermore, to better train the model, we designed a training strategy to adjust the contribution of each loss. It is known that the CycleGAN model has three types of loss: (1) consistency loss ($L_{cyc}$), (2) identity loss ($L_{id}$), and (3) adversarial loss ($L_{adv}$). Generally speaking, the consistency loss mainly contributes to conversion; the identity loss primarily contributes to the preservation of the original features; while the adversarial loss maintains the integrity of the generated sample close to the real one. The total loss of the adopted CycleGAN is expressed in Equation (5), where the α and β parameters are adjusted during training.

(5) $$L=L_{adv}+\alpha L_{cyc}+\beta L_{id}$$

The training strategy is presented in Algorithm 1. The α and β parameters were initialized to 1. After 65% of the epochs during the training procedure, β was adjusted to 0.5 while keeping α at 1, so that the model could pay more attention to the conversion. Meanwhile, the learning rate was slightly decreased.
**Algorithm 1** Training strategy1:lr=2e−4; optimizer=Adam(lr,beta_1=0.5);2:α=1; β=1;3:**for** epoch in epochs **do**4:    **if** epoch>(epochs × 65%) **then**5:        α=1, β=0.5;6:        lr+=−5e−8;7:    **end if**8:**end for**9:α=1, β=1;

Table 3 presents the objective results of the CycleGAN with curriculum learning and the plain CycleGAN [47]. *N* indicates the neutral emotion; *P* indicates positive, while LS indicates low spirit. The mel-cepstral distortion (MCD) was adopted to evaluate the spectrogram conversion as in Equation (6) where $MECPs_{i}^{t}$ indicates the target mel-cepstrum, while $MECPs_{i}^{c}$ represents the converted one. The root mean squared error (RMSE) is used to evaluate F0 conversion as in Equation (7); $F0_{i}^{t}$ and $F0_{i}^{c}$ represent the target and converted F0 features, respectively. Both evaluation metrics prefer lower values. From the results, it is observed that CycleGAN with curriculum learning achieves better performance in converting the spectrogram and F0, in comparison to the baseline of the plain CycleGAN.

(6) $$MCD\left[db\right]=\frac{10}{\ln10}\sqrt{2\sum_{i=1}^{24}{\left(MECPs_{i}^{t}-MECPs_{i}^{c}\right)}^{2}}$$

(7) $$RMSE=\sqrt{\frac{1}{L}\sum_{i=1}^{L}{\left(F0_{i}^{t}-F0_{i}^{c}\right)}^{2}}$$

**Figure 2:**
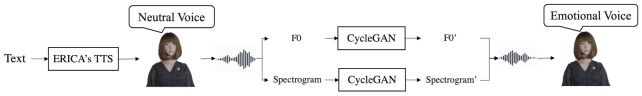
Flow of obtaining emotional voice.

Figure 2 depicts the procedure of generating ERICA’s emotional voice. First, ERICA’s built-in text-to-speech (TTS) system was used to convert text to speech. Note that, ERICA’s TTS can only generate neutral voice. To obtain emotive speech, we used the trained emotional voice conversion model in the CycleGAN framework to prepare low-spirit voice and positive voice to convey empathy and encouragement, respectively. Note that, in this study, the focus is on the effects of emotional expression of audio modality. That is, emotional facial expressions and gestures of ERICA are not designed. Given the prepared ERICA’s emotional samples, we invited ten subjects (M=23.1, SD=3.1) to conduct pre-hoc evaluation to check whether the emotion is successfully converted using the mean-opinion-score (MOS) with five-level Likert scale. −2 indicates low-spirit emotion; 0 indicates neutral emotion; and the 2 indicates positive emotion.

**Figure 3:**
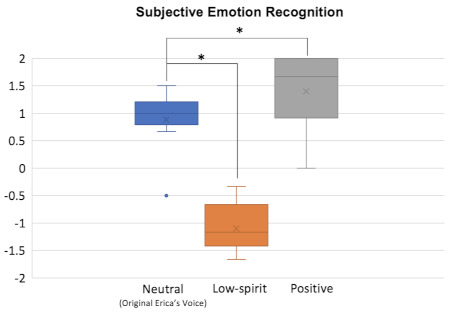
Emotion evaluation of the original and converted emotion. * indicates the found significant difference.

Figure 3 presents the subjective evaluation results analyzed with pair-wise student-*t* test. There were significant differences between the original voice and converted low-spirit voice (*t*(9) = 1.9, *p* < 0.05), as well as the original voice and converted positive voice (*t*(9) = 8.91, *p* < 0.05). These results suggested that the emotion was successfully converted by the model, and subjects could distinguish the difference in emotions.

### 4.2. Measurements

We designed questionnaires to allow subjects to evaluate the robot’s ability to express emotions with the audio modality. Furthermore, users’ perception of ERICA’s empathy and the ability to convey encouragement were measured. In addition, the willingness of users to talk to ERICA when depressed was evaluated.

The questionnaire designed to evaluate ERICA’s emotional expression and performance is provided below: 


***- Emotional expression:***
I can feel the sad feeling of ERICA’s voice.I can feel the positive spirit of ERICA’s voice.
***- Evaluation on ERICA:***
ERICA is empathetic.ERICA is encouraging me.I would like to talk to ERICA when I am feeling down.


To evaluate the personality of ERICA, ten item personality inventory (TIPI-J) [23] was adopted as in previous research [49,50,51] containing the following questions. In the questionnaire, all the questions were evaluated with a 7-level Likert scale. 


***- Extroversion:***
ERICA seems to be the active, outgoing type.ERICA seems to be the reserved, quiet type.
***- Agreeableness:***
ERICA is prone to complaining and getting into trouble with others.ERICA is kind and cares about other people.
***- Conscientiousness:***
ERICA seems to be a firm, self-disciplined type.ERICA seems to be the sloppy, careless type.
***- Neuroticism:***
ERICA seems to be the type that worries and frets easily.ERICA seems to be the calm, emotionally stable type.
***- Openness:***
ERICA likes new things and has unique ideas.ERICA is uninspired and mediocre.


### 4.3. Hypotheses

It is expected that ERICA’s emotional changes through voice can enhance the perception of empathy and shape ERICA’s positive personality to some extent. According to the questionnaire, the hypotheses are as follow:

- H1: The proposed method reinforces ERICA’s ability to express emotion with voice, and obtain a higher score in terms of emotional expression;
- H2: The proposed method improves the perception of empathy and encouragement;
- H3: When feeling down, people prefer to talk with the ERICA equipped with the proposed method;
- H4: Compared to the neutral voice, the emotional expression of the voice can better shape the extroversion and openness of the robot to some extent.

## 5. Experiment

### 5.1. Scenarios and Conditions

In the designed three short human–robot conversion transcripts, ERICA asked about the user’s recent situation and shares similar experiences of others. The transcripts included the topics of job hunting, corona life, and loving relationship, with the aim of having the user share the current dilemma, while ERICA tried to express empathy and encouragement for the user. Since the conversation was set up to share the user’s predicament, a sad (low-spirit) voice was set to share the similar history of others in presenting ERICA’s semantic and emotional comprehension, namely, the empathy ability. After that, the positive voice of ERICA was used to provide encouragement to the user. As a comparison, the neutral voice of ERICA’s utterances was used for the control condition. Table 4 demonstrates an example of the dialogue transcript, in which EXP. indicates the experimental condition, while CON. indicates the control condition.

### 5.2. Procedures and Subjects

First, a user was invited to talk with ERICA according to transcripts, and the conversation was recorded. Subsequently, we created a subjective evaluation questionnaire based on the adopted measures. The questionnaire contained two conditions, the experimental condition and control condition, each having three conversation videos. Subjects rated ERICA’s performance after watching the recorded video according to each question. In the experiment, the within-subject design was used, namely, each subject evaluated both the emotional ERICA and neutral ERICA. Sixteen subjects (Male = 10, Female = 6, M=23.94, SD=2.61) were invited as raters to participate in the questionnaire-based evaluation by watching the recorded conversation video.

### 5.3. Results and Discussion

After collecting the subjective evaluation data, we used the paired student *t*-test to analyze the results with alpha set to 0.05. Additionally, Cohen’s D was calculated and annotated as *D* when reporting the results. Moreover, as some previous studies suggested that gender differences need to be considered when robots take on the role of a self-disclosing listener or companion [52,53], we investigated the gender effects in the designed human–robot comforting dialogue.

Regarding emotional expression, which checked manipulation in this experiment, the results show that, after implementing the emotional voice of ERICA, the experimental condition (M=4.75, SD=1.12) strengthened ERICA’s ability to express low spirit (p<0.001,t(15)=3.59,D=1.27) in contrast to the control condition (M=3.19, SD=1.33), but only incrementally on positive expression without significant difference. This suggested that the manipulation of ERICA’s positive expression did not completely succeed.

**Figure 4:**
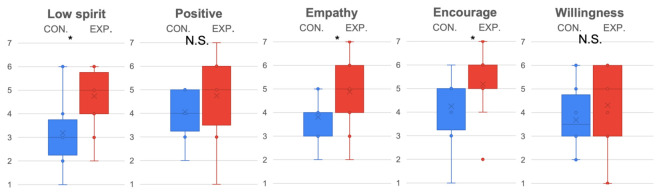
Emotion expression and evaluation of ERICA evaluated by all subjects. * indicates the found significant difference, while N.S. indicates there is no significant difference.

Considering the evaluation of ERICA (see Figure 4), it was determined that the experimental condition (M=4.88, SD=1.45 ) better represented ERICA’s empathy ability (p<0.01,t(15)=2.53,D=0.91) than the control condition (M=3.81, SD=0.83). Additionally, subjects rated that the encouragement (p<0.05,t(15)=2.22,D=0.78) was better conveyed in the experimental condition (M=5.19, SD=1.17) compared to the control condition (M=4.25, SD=1.24).

**Figure 5:**
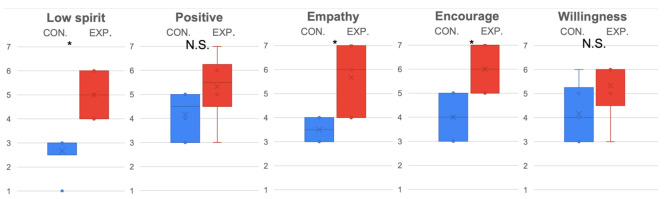
Emotion expression and evaluation of ERICA evaluated by female subjects. * indicates the found significant difference, while N.S. indicates there is no significant difference.

**Figure 6:**
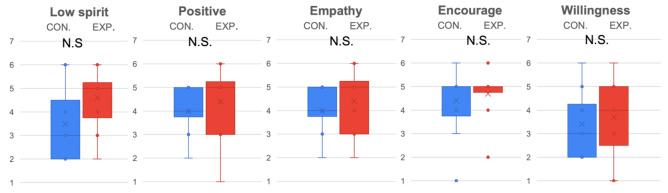
Emotion expression and evaluation of ERICA evaluated by male subjects.

The evaluation results of female (see Figure 5) and male (see Figure 6) subjects were further compared separately. Because only partial data were reused, a Bonferroni correction with 3 degrees was adopted to lower the alpha (α=0.05/3). From the statistical results, it was determined that females were more sensitive to ERICA’s low spirit expression (p<0.05/3,t(15)=4.72,D=2.43) in the experiment condition (M=5, SD=0.89) than in the control condition (M=2.67, SD=0.82). Significant differences were observed for the experiment condition (M=5.67, SD=1.37) (M=6, SD=0.89) and control condition (M=3.5, SD=0.55) (M=4, SD=0.89) in terms of empathy (p<0.05/3,t(15)=3.61,D=1.86), and encouragement (p<0.05/3,t(15)=3.87,D=1.99), while males only displayed marginal statistical significance for the experiment condition (M=4.6, SD=1.26) and control condition (M=3.5, SD=1.51) in terms of perceiving ERICA’s low spirit voice significantly (p=0.0471,t(15)=1.77,D=0.92). In addition, it is worth mentioning that the trend of female subjects was more significant for the experiment condition (M=5.33, SD=1.21) and control condition (M=4.17, SD=1.17) in terms of preferring to chat with ERICA with an emotional voice (p=0.0602,t(15)=1.7,D=0.91).

**Figure 7:**
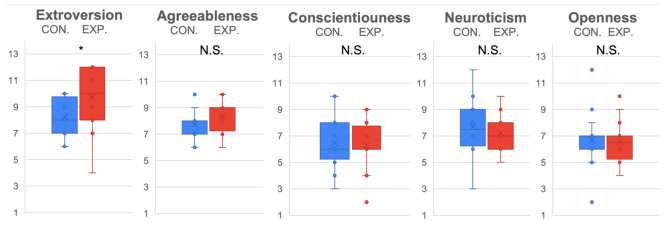
Personality evaluation of ERICA with Big Five by all subjects. * indicates the found significant difference, while N.S. indicates there is no significant difference.

**Figure 8:**
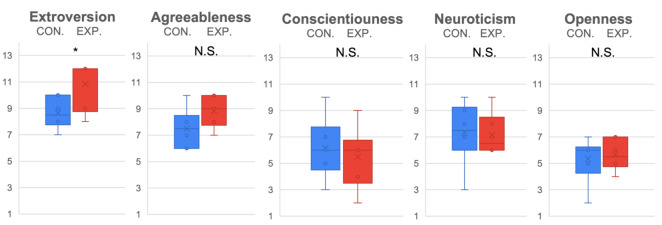
Personality evaluation of ERICA with Big Five by female subjects. * indicates the found significant difference, while N.S. indicates there is no significant difference.

**Figure 9:**
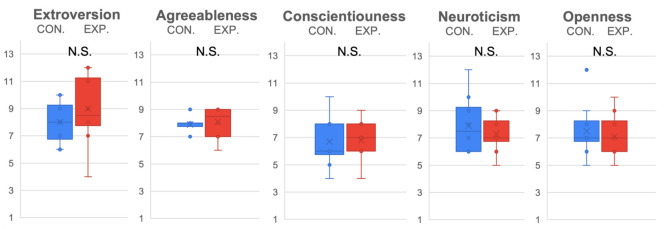
Personality evaluation of ERICA with Big Five by male subjects.

In the personality evaluation of ERICA, as in Figure 7, the emotional voice made ERICA more outgoing (p<0.05,t(15)=2.06,D=1.06) in the experiment condition (M=9.69, SD=2.41) than in the control condition (M=8.25, SD=1.39). However, when male subjects and female subjects were separated for analysis, it was determined that the significant difference from the experiment condition (M=10.83, SD=1.83) and control condition (M=8.67, SD=1.21) of ERICA’s extroversion mainly originated from females (p<0.05/3, t(15)=2.41,D=1.24), whereas males only expressed a similar tendency without significant difference (see Figure 8 and Figure 9). In addition, when equipped with an emotional voice, subjects tended to evaluate ERICA with higher agreeableness (p=0.0555,t(15)=1.64,D=0.85) in the experiment condition (M=8.38, SD=1.15) than in the control condition (M=7.75, SD=1).

Based on the statistical results, the hypotheses were corroborated to some extent. For Hypothesis 1, the proposed emotional conversion model only enhanced the expression of the low spirit of ERICA, while the positive expression was not strengthened. It suggested that the manipulation of positive expression might not completely succeed with our method. This was possibly because ERICA’s original speech had already been positive, so that the difference of the converted positive voice was not really distinguishable. This also implied that the effects of the proposed strategy might mainly result from the low-spirit expression. Therefore, the emotional conversion model needs to improve the performance of converting positive speech. In future work, more modalities will be combined to generate the robot’s emotions. For Hypothesis 2, the results show that our approach successfully improved the perception of empathy in conveying encouragement. Considering the failure on controlling positive speech, ERICA was better able to convey encouragement because of the expression of low-spirit, which enhanced the perception of empathy. For Hypothesis 3, no significant difference was found in the results, but only slight non-significance for female subjects who talked with the ERICA equipped with emotional speech. The current strategy and dialogue content were quite simple and the dialogue had fewer iterations. The purpose of comfort could not really be achieved. We plan to go deeper into the investigation of HHI, and acquire additional insights to enrich the way the robot responds and expresses its emotions, especially facial expressions and body movements. For Hypothesis 4, our approach helped the robot to demonstrate extroversion, but failed to significantly present the openness. This was because the dialogue had fewer iterations, and the robot was not able to share additional experiences. Future work is going to improve the dialogue strategy and extend the dialogue, so as to provide more chance to the robot to present experiences.

## 6. Where Next

In this section, we acknowledge the limitations of this work along with plans to address them in future works.

### 6.1. The Effect of Experience Sharing

This work validated the effects of the combination of emotional speech and experience sharing in human–robot comforting dialogue. However, which of these two factors provided the major contribution to enhancing the perception of empathy remains to be verified. Furthermore, this work adopted the experience sharing strategy of previous works [15,16,17], which could facilitate the human–robot interaction. What needs to be discussed is whether a robot sharing an experience similar to other people could demonstrate perspective-taking in the human–robot comforting dialogue. One possible future direction is to systematically examine the effects of experience sharing by adding another independent condition, in which the robot only talks about the current user without mentioning other people’s experiences.

### 6.2. The Design of Robots’ Emotional Responses

In the experiment condition, ERICA responded to user’s emotions with similar affective expressions to reveal insight from the findings of HHI [34,48]. However, these results were not the emotional responses in the conversational context of Japanese. Although positive results were obtained by using this strategy, the appropriateness needs to be verified and further improved. Therefore, Japanese emotional conversation data should be collected to analyze how Japanese people conduct emotional interactions. Moreover, our aim is to explore how robots can provide emotional comfort to people. In many of the current studies, including this one, robots are mimicking people performing comforting behaviors in HHI. Perhaps, there could be a way of comforting that is unique to robots.

### 6.3. Multi-Modality Emotional Expression

This study only implemented the audio-modality emotional expression for ERICA, which was also a preliminary study for examining the emotional voice conversion model. This robot platform is able to perform multi-modality (i.e., facial expression, gesture) emotional expressions. Therefore, future work will generate ERICA’s multi-modality emotional expression for the human–robot comforting dialogue. It is also assumed that the multi-modality method can enhance the perception of positive expression. Future investigations should be based on the two dimensional emotion framework (negative and positive, low and high arousal) to offer better generalizability.

### 6.4. Gender Effects

From the experimental results, some gender effects of the designed human–robot comforting dialogue were observed preliminarily; however, the sample size was relatively small. The next step is to increase the number of participants to confirm if this phenomenon is applicable on a larger scale to more numerous subjects. After confirming this, the emotional behaviors of the robot can be modified according to the discovered gender effect to design strategies more applicable for men and women.

### 6.5. Experiment with Practical Comforting Interaction

There were some technical difficulties in conducting the practical human–robot comforting dialogue in real-life. Thus, video-based evaluations were used with a third-party perspective in this work. Even though the scripts were designed with recent common topics, such as the impact of the epidemic, bad experiences in job hunting, and quarrels with the partner, it is only in real scenarios that the improvements provided by our method can be better validated. Therefore, in our future work, first, the technical problems (making people willing to share their predicament with robots) will be solved in human–robot comforting, and then a practical comforting interaction experiment will be conducted.

## 7. Conclusions

This paper introduced a preliminary study on human-robot comforting conversation. The main contributions are summarized as follows: (1). CycleGAN-based emotional voice conversion model was trained with curriculum learning on a Japanese emotional speech dataset to obtain ERICA’s emotional voice; (2). Emotion and experience sharing strategies were combined to let ERICA present an understanding of the user’s utterance and emotion status to enhance the perception of empathy and personality. The experimental results showed that the proposed method might be able to help ERICA better convey encouragement and low spirits in emotion, empathy, the personality of extroversion, especially for females. In future work the objective is to construct a practical emotional experience sharing dialogue system for human–robot interaction. The implementation of our emotional voice conversion model and experiment videos can be found at https://github.com/CZFuChason/emotional-voice-conversion-with-CycleGAN (accessed on 9 December 2021).

## Figures and Tables

**Table 1:** The percentage (%) of emotional responses between speaker and interlocutor in human–human interaction (IEMOCAP). Bold numbers represent the highest value; italicized numbers represent the second highest value.

		Interlocutor’s Emotional Responses						
		Happy	Sad	Frustrated	Surprise	Anger	Excited	Neutral
Speaker’s status	Happy	**69.81 **	0.71	0.24	2.89	0	16.98	*10.38 *
	Sad	0.32	**88.29**	*5.59*	0.21	0.54	0.21	4.30
	Frustrated	1.40	3.85	**72.22**	0.41	*13.86*	0	9.52
	Surprise	10.26	1.28	10.26	**39.74**	2.56	*24.36*	8.97
	Anger	0	0.97	38.37	0.19	**57.95**	0.58	1.94
	Excited	9.42	0.11	0.11	1.71	0	**81.69**	6.00
	Neutral	2.77	2.48	*10.50*	1.46	0.29	4.38	**78.05**

**Table 2:** The percentage(%) of emotional responses between speaker and interlocutor in human–human interaction (MELD). Bold numbers represent the highest value; italicized numbers represent the second highest value.

		Interlocutor’s Emotional Responses						
		Disgust	Joy	Neutral	Angry	Fear	Sadness	Surprise
Speaker’s status	Disgust	*16.10 *	7.20	**36.44 **	13.98	2.96	9.75	13.56
	Joy	2.26	*33.63*	**38.95**	7.50	2.33	4.66	10.67
	Neutral	2.29	*14.89*	**55.53**	8.30	2.19	5.00	11.80
	Angry	2.40	10.72	**35.87**	*31.46*	3.01	7.14	11.16
	Fear	1.65	13.22	**41.32**	*13.64*	11.57	7.43	11.16
	Sadness	3.48	10.76	**34.11**	10.43	2.65	*24.34*	14.24
	Surprise	2.58	15.38	**45.30**	10.41	2.76	7.09	*16.48*

**Table 3:** Objective comparisons for emotional voice conversion.

	MCD			RMSE		
Models	N→P	N→LS	Avg.	N→P	N→LS	Avg.
CycleGAN-CL (*ours*)	18.94	17.56	18.25	135.11	84.42	94.77
CycleGAN [47]	20.28	17.88	19.08	137.64	89.97	113.81

**Table 4:** An translated example of dialogue transcripts (the original script was in Japanese).

	Utterance	Emotion (EXP.)	Emotion (CON.)
ERICA	Hi, Yuki, meet you again, how is it going?	Neutral	Neutral
User	Due to Covid-19, I haven’t been able to go out for about a week, I felt a little down.	Low spirit	Low spirit
ERICA (experience sharing)	That is tough. Delina once told me that she had to wear a mask every time she left the apartment, which was quite inconvenient.	**Low spirit **	**Neutral**
User	That’s right. I do not even want to go out.	Low spirit	Low spirit
ERICA (encouragement)	It is better to go out for a walk sometimes to refresh yourself! With a mask on.	**Positive**	**Neutral**

## Data Availability

IEMOCAP dataset: https://sail.usc.edu/iemocap/ (accessed on 9 December 2021); MELD dataset: https://github.com/declare-lab/MELD (accessed on 9 December 2021); Japanese emotional speechdataset: https://aclanthology.org/2020.lrec-1.62.pdf (accessed on 9 December 2021).
